# The Feasibility of the Arabic Version of Ages and Stages Questionnaire 3 to Identify Preterm Infants at Risk of Developmental Delays in Saudi Arabia

**DOI:** 10.3390/pediatric17050105

**Published:** 2025-10-13

**Authors:** Turki Aljuhani, Waad Aljurayyad, Ibrahim F. Almudayfir, Ruyuf M. Alhassan, Monerah I. Alharran, Razan A. Aloushan, Reem S. Alsaleem, Nassar M. Althunayyan, Reem A. Albesher

**Affiliations:** 1 Department of Occupational Therapy, College of Applied Medical Sciences, King Saud Bin Abdulaziz University for Health Sciences, Riyadh 11481, Saudi Arabia; juryyadw@ksau-hs.edu.sa (W.A.); almudayferi@ksau-hs.edu.sa (I.F.A.); 421210137@ksau-hs.edu.sa (R.M.A.); 421210282@ksau-hs.edu.sa (M.I.A.); 421210078@ksau-hs.edu.sa (R.A.A.); 421210119@ksau-hs.edu.sa (R.S.A.); 2 King Abdullah International Medical Research Center, Riyadh 11481, Saudi Arabia; althunyyanna@mngha.med.sa; 3 Department of Rehabilitation, King Abdullah Specialized Children’s Hospital, Ministry of National Guard Health Affairs, Riyadh 11426, Saudi Arabia; 4 Department of Rehabilitation Sciences, College of Health and Rehabilitation Sciences, Princess Nourah bint Abdulrahman University, Riyadh 11671, Saudi Arabia; raalbesher@pnu.edu.sa

**Keywords:** preterm, screening tools, developmental delays, Ages and Stages Questionnaire

## Abstract

Objectives: Preterm infants are at higher risk for developmental delays (DDs) and long-term complications compared with term infants. With the high prevalence of preterm births in Arabic-speaking countries, an Arabic-language screening tool is crucial. The aim of the study is to examine the feasibility of utilizing an Arabic version of the Ages and Stages Questionnaire, Third Edition (A-ASQ-3), at 4 months corrected age (CA). Methods: Infants born at or after 28 weeks of gestational age were recruited in this longitudinal study. A total of 48 infants underwent the developmental assessment at 4 months CA using the A-ASQ-3. The primary outcome was identifying the infants at risk for DDs. Descriptive statistics, t-tests, and Firth’s logistic regression were used for analysis. Results: Of the 48 infants, 37 (77.1%) had a DD at in least one of the five A-ASQ-3 domains at 4-months CA. None of the risk factors assessed in this study were associated with a high risk of DDs among preterm infants. Conclusions: The A-ASQ-3 is a feasible tool for identifying infants at risk for DDs at 4 months CA. This finding underscores the need for early screening and tailored intervention programs for preterm infants in Saudi Arabia. The A-ASQ-3 can help identify infants at high risk of DDs and enable prompt referral to healthcare providers.

## 1. Introduction

Preterm birth is a worldwide concern, with recent reports estimating that 15 million babies are born preterm globally each year, predominantly in low- and middle-income countries [1,2]. In Saudi Arabia, a recent study estimated that 8.4% of all births are preterm, with a similar percentage of 9% identified in another study [3,4]. Preterm birth, defined as babies born alive before 37 full weeks of gestation, is usually associated with adverse neonatal and maternal outcomes [5]. Although moderate and late preterm infants have a better survival rate compared to extremely preterm infants, all preterm infants are at risk of developmental delays (DDs) [6]. In recent years, the cut-off of 37 weeks for preterm and term birth have been consider inconstant [7]. The World Health Organization (WHO) dived preterm based on gestational age (GA) into the following categories: extremely preterm (<28 weeks), very preterm (28–<32 weeks), and moderate or late preterm (32–<37 weeks GA) [5].

Multiple challenges are faced in applying early screening tools to identify infants at risk of DD in Saudi Arabia. One of the major challenges is the limited number of culturally sensitive developmental screening tools that are validated for children aged < 5 years in different cultures [8]. Another challenge is the lack of using standardized tools such as the General Movement Assessment for accurate and early diagnosis of DDs [9]. For example, one study in Saudi Arabia found that 60% of healthcare providers played a role in identifying conditions that maybe associated with DDs. The healthcare provider did not consistently check for normal development or DDs in infants and toddlers [10]. Thus, the need for an Arabic-language early screening tool is crucial.

Moderate or late preterm infants are shown to exhibit higher rates of DDs than term infants [11]. Moreover, recent studies have further confirmed a high incidence of DDs in the moderate and late preterm groups [12,13]. DDs can manifest in a single domain, such as gross motor, fine motor, language, social–emotional, or problem-solving skills, or across multiple domains, and can be present at 2 years old. When delays are observed in two or more domains before the age of 5 years, the child is considered to have a global DD [14]. A study conducted in Saudi Arabia found that 17.4% of preschool children have DDs; however, the restricted scope and sample size of the study limit its generalizability [15]. The studies discussed above highlight the need for early detection of and intervention programs for infants at risk of DD.

Developmental screening is crucial for ensuring the optimal development of children and the timely detection of DDs. The American Academy of Pediatrics (AAP) emphasizes the early identification of developmental disorders, which impact both the child and their family. The AAP recommends standardized developmental screening for all toddlers at 9, 18, and 30 months of age [16]. Standardized screening measures can assist in the prompt detection of DDs, thereby enabling early intervention [17,18,19,20]. The AAP policy statement details various screening methods and algorithms, including standardized questionnaires for parents such as the Parental Evaluation of Developmental Status, Ages and Stages Questionnaire (ASQ), and Child Development Inventory. The ASQ can be used as a screening tool to identify infants at risk of delays with a good psychometric property [21,22]. The advantages of these assessments over direct assessment completed by professionals is that the parent-completed screening questionnaires allow for reduced costs, increased accuracy, and regular reporting on a child’s development [16,23]. However, the APP policy applies for infants and young children without identified risk or clear developmental issues. Infants who are born with identified risk (i.e., low birth weight and/or preterm) should be screened and monitored at a younger age [24,25]. In addition, studies showed that signs of DD at 4–8 months can assist as an indicators of later outcomes, thus emphasizing the need for administering screening tools as early as 4 months [26,27].

The ASQ screening was developed in response to the growing demand for early and accurate identification of children with DDs or developmental disorders. The ASQ screening system has been validated in multiple languages, including Arabic. The third edition (ASQ-3), revised in 2009, contains 21 questionnaires for infants aged 2–66 months. Research has shown that ASQ-3 has good psychometric properties, with 75% sensitivity and 81% specificity, and modest agreement with the Bayley-III scale in detecting DDs in preterm infants at 8, 18, and 30 months corrected age (CA) [28]. The ASQ-3 was first adapted into Arabic as the A-ASQ and has shown culture-sensitive and acceptable internal consistency and reliability in younger age groups [29,30]. Moreover, the A-ASQ-3 demonstrated adaptable reliability and validity when measured in 491 infants and toddlers aged 4–33 months [28]. To our knowledge, only one study has used the A-ASQ-3 to screen preterm infants in Saudi Arabia, finding that 42.6% of 62 infants had at least one DD at 18, 20, and 24 months CA [19].

In the context of Saudi Arabia, there is a lack of utilizing early screening or assessment tools. This is due to the lack of training of healthcare providers (i.e., not checking normal development or DD in infants and toddlers) and the limited availability of family-oriented assessments that are culture-adapted. Considering that the A-ASQ-3 has been applied in Saudi Arabia only to a limited extent, our study has two aims: (1) to examine the feasibility of the A-ASQ-3 as a screening tool for preterm infants at risk for DDs at 4 months CA and compare between infants at high risk of DD and infants with no risk of DD and (2) to investigate the association between being at risk for DDs and potential risk factors. The current study does not aim to validate the use of the A-ASQ-3 nor provide diagnostic accuracy of the A-ASQ-3.

## 2. Materials and Methods

### 2.1. Design

This feasibility longitudinal study was conducted at the King Abdullah Specialized Children’s Hospital (KASCH) in Riyadh, Kingdom of Saudi Arabia, between 1 October 2023 and 31 June 2024, to test the acceptability of the A-ASQ-3. Preterm infants were recruited from the Neonatal Intensive Care Unit (NICU). Eligibility criteria included preterm infants born at or after 28 weeks of GA at NICU and those who were receiving continuous care at pediatric outpatient clinics whose parents were able to read and speak Arabic. The exclusion criteria encompassed infants with conditions such as hypoxic–ischemic encephalopathy, known brain injury, genetic diagnoses, intraventricular hemorrhage (IVH) grades 3 and 4, and whose parents were unable to read and speak Arabic.

Infants were also classified, based on the prevalence of respiratory distress syndrome (RDS), into three categories: those with RDS classified as Chronic Lung Disease (CLD), those without RDS, and those with resolved RDS in the NICU. Furthermore, infants were categorized into three subgroups based on birth weight in grams, extremely low birth weight (<1000 g), very low birth weight (<1500 g), and low birth weight (<2500 g), according to WHO categories. Risk factors investigated in this study were length of stay, birth weight, and risk of CLD.

**Figure 1:**
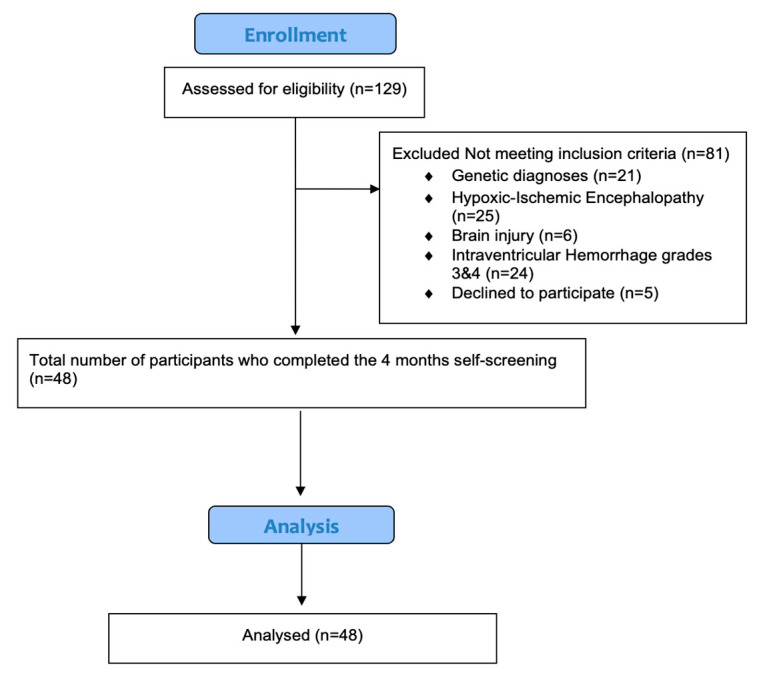
Flowchart of the recruitment process of the study.

Using the Epi Info sample size calculator and data, we calculated the required sample based on a power of 80%, a 95% confidence interval (CI), and a 50% prevalence of DDs in preterm infants. The calculation determined a minimum sample size of 32. Non-probability convenience sampling was employed to recruit parents of preterm infants meeting the inclusion criteria. This sampling technique was used to avoid difficulties in reaching out to parents and to allow the consent of the participants. A total of 129 infants were assessed for the study eligibility; of these, 48 completed the 4 months CA assessment Figure 1.

Ethical considerations were paramount, with data collection commencing after institutional review board approval was obtained from the King Abdullah International Medical Research Center (approval number: IRB/1870/23). Informed consent was obtained from the families, and they were assured of confidentiality and privacy in data handling.

### 2.2. Arabic Ages and Stages-3 Assessment

The A-ASQ-3 assesses five developmental domains—communication, problem-solving, personal–social, gross motor, and fine motor skills—with a total score of 60 for each domain. Depending on the behavior described in each item under each domain, there were three possible responses: “yes” (10 points), “sometimes” (5 points), and “not yet” (0 points). The total score for each domain was calculated by adding the scores of the six items within the domain. Cut-offs were set at two standard deviations (SDs) above or below the mean. The main goal of the A-ASQ-3 was to identify DDs in children. A specific objective of the research included assessing the feasibility of the A-ASQ-3 for early DD detection, which was defined as an A-ASQ-3 score of less than one SD (1SD) for “mentoring zone” and less than two SDs (2SD) for “high risk of DD” in one or more domain. The A-ASQ-3 is a valid and simple screening tool used to differentiate and identify DDs; it has a sensitivity and negative predictive value approaching 100% and an acceptable specificity of 76% [30]. The A-ASQ-3 is recommended as an effective screening tool for routine monitoring of low-risk children due to its psychometric properties as well as cultural sensitivity [29]. It has modest agreement with the Bayley-III scale (r = 0.56) in detecting DDs in preterm infants [30].

The electronic health records (BestCare, ezCareTech, Seoul, Republic of Korea) system was used to extract data on the identified infants. The A-ASQ-3 was administered in two phases: the first phase involved interviewing parents via phone calls, and the second phase was completed through an online survey. Parents filled out the questionnaire individually or with assistance, capturing developmental assessments. For this study, data collection was performed at 4 months CA. Calculation of the scores was completed by the research team.

### 2.3. Statistical Analysis

Data were entered into SPSS version 27. Frequency and percentages were used to represent the qualitative data. Quantitative data were assessed using an independent *t*-test or Pearson’s chi-square test of independence (as appropriate) and presented as means and SDs. The A-ASQ-3 scores of the two groups (high risk vs. no risk) were compared using analysis of covariance while controlling for the GA of the infants. Firth’s logistic regression was employed as a standard approach for analyzing binary outcomes with small samples to determine significant predictors, odds ratios, and a 95% CI. While this method reduces bias in the maximum likelihood estimates of coefficients, it introduces a bias toward one half of the predicted probabilities. A *p*-value of <0.05 was considered statistically significant.

## 3. Results

### 3.1. Participants

A total of 48 infants were included in this study, all of whose parents completed the A-ASQ-3 by 12 or 13 weeks CA. Results from the A-ASQ-3 showed that 11 infants (22.9%) showed typical development at the 4-month CA screening, whereas 37 (77.1%) were found to be at high risk of DDs (<2SD) in one of the five domains. The neonatal characteristics of infants at high risk of DDs and those at no risk of DDs are shown in Table 1. There were no statistically significant differences between the groups. The mean length of stay was higher in the group with no risk of DDs (52 days) than in the group with high risk (39 days).

Most infants at high risk of DDs (n = 37) were female (n = 21), had resolved RDS (n = 32), were very preterm (n = 25), and had very low birth weight (n = 23). The prevalence of periventricular leukomalacia (PVL) (4 vs. 1) and IVH grades I and II (12 vs. 6) was higher in infants at high risk of DDs compared with those at no risk.

### 3.2. A-ASQ-3 Scores and Risk of Developmental Delays

Each of the five domains of the A-ASQ-3 were examined, with a total of 48 infants. For the communication domain, no significant difference was found between the two groups (Table 2). However, the gross motor domain showed a significant difference (*p* = 0.004), with scores of 31 infants showing no risk and 17 infants at high risk. A similar finding was reported in the fine motor skills domain (*p* < 0.001), with half (24) of the infants with no risk and the other half (24) being those with high risk. Both the problem-solving and personal–social domains also demonstrated significant differences (*p* < 0.001). The respective numbers for the no-risk group were 28 and 17, and those for the high-risk group were 20 and 31 (Table 2).

Furthermore, each of the five domains of the A-ASQ-3 accounted for a total of 60 points. The gross motor domain showed a significant difference (*p* = 0.004), with a mean score of 52.2 for infants without risk and 34.7 for infants at high risk. A similar finding was reported in the fine motor skills domain (*p* < 0.001), with means of 46.4 for infants with no risk and 17.6 for those with high risk. Both the problem-solving and personal-social domains also demonstrated significant differences (*p* < 0.001). The respective means for the no-risk group were 56.2 and 42.9, and those for the high-risk group were 26.7 and 17.9 (Supplementary Materials Table S1). A total of 12 (32.4%) infants in the high-risk group showed DDs in one domain only. Most infants had DDs in more than one domain (25 infants, 67.6%); of these, five infants in the high-risk group showed DDs in all five domains of the A-ASQ-3.

### 3.3. Risk Factors Associated with Developmental Delays

Binary logistic regression analysis revealed that none of the variables, including length of stay, birth weight, and risk of RDS, were significantly associated with the increased risk of DDs in our cohort (Table 3).

## 4. Discussion

This feasibility study examined the use of the A-ASQ-3 for screening DDs in preterm infants at 4 months CA. While, the aim of the study was to test the feasibility of the A-ASQ-3, we found a high prevalence (77.1%) of DDs in at least one of the five domains assessed in preterm infants; our findings were notably higher than recent studies reporting prevalence rates of 26%, 43%, and 42.6% in similar populations [19,31,32]. Compared with Al-Hindi et al.’s study on toddlers (18–24 months), this study reported higher prevalence rates across all domains: communication (14.6% in this study vs. 11.5% reported by Al-Hindi et al.), gross motor (35.4% vs. 11.5%), fine motor (50.0% vs. 19.7%), problem-solving (41.7% vs. 19.7%), and personal–social skills (64.6% vs. 23%) [19]. This discrepancy may be attributed to the younger sample and population-specific factors, as early signs of DDs may naturally resolve over time or require further interventions for optimal development. Yet, a local study in Saudi Arabia reported that 75% of infants had DD since birth [33]. These inconsistent findings are attributed to the different timepoints of screenings and possibly the cut-off scored using the screening tools.

Furthermore, some studies have questioned the effectiveness of the ASQ-3 screening in identifying general DDs, and particularly communication DDs, when performed on infants under the age of 9 months [21,32]. The consistent distribution pattern of DDs across domains, which were observed in both studies despite age differences, suggests persistent challenges in specific areas of development among preterm infants in Saudi Arabia. This pattern, characterized by certain domains consistently showing higher or lower percentages of DDs, may indicate the effectiveness—or lack thereof—of current early intervention programs in the country [19]. However, the findings of this study underscore the importance of routine, early developmental screening for preterm infants in Saudi Arabia, which can potentially allow for more effective and less expensive treatment during preschool years, thereby improving long-term outcomes [34,35]. Additionally, early screening can help parents communicate more effectively with their healthcare providers and seek additional information or tests if required [36].

As reported by Almalki et al., early intervention services in Saudi Arabia may require further improvement in the medical, familial, and educational dimensions [37]. This finding aligns with our observations, indicating that if interventions are not tailored to address specific developmental domains, patterns of DDs might persist across age groups [37]. To address this issue comprehensively, longitudinal studies are crucial. Such studies can allow researchers to track developmental trajectories, evaluate long-term intervention effectiveness, and identify critical periods for targeted support in preterm infants in the country.

Significant differences were observed between the no-risk and high-risk groups across the gross motor, fine motor, problem-solving, and personal–social domains, as measured by the A-ASQ-3. These differences, based on the 2SD threshold, were expected given the grouping criteria, with the high-risk group demonstrating lower mean scores in each domain. This finding is in line with the ASQ-3′s design as a screening tool and supports its ability to differentiate between infants at varying levels of developmental risk [28]. Notably, while these differences are statistically significant, their clinical significance and predictive value for long-term outcomes are not conclusive and require further investigation through longitudinal studies [22,38]. In terms of the risk factors of DDs, our results suggested no correlation between our current cohort and any of the assessed risk factors, while observed differences between the infants with no risk of DD and infants with risk of DD can be shown in weight at birth. For example, a higher percentage of infants with very low birth weight (VLBW) was found in the high-risk group (85.2%) than in the low-risk group (14.8%), yet in infants with extremely low birth weight (ELBW) a slightly higher percentage of infants was found in the no-risk group than in the high-risk group (57.1% vs. 42.9%), thus suggesting that birth weight alone is not a determining factor for DDs. This finding contradicts those of some previous studies, which have found an association between ELBW and an increased risk of DDs. However, the effects of birth weight on development may not be fully apparent at the early age of our sample population, which may contribute to the aforementioned discrepancy. Research on the growth patterns of preterm and small-for-gestational-age children has shown that while preterm infants exhibit rapid postnatal growth, they still have lower height-for-age z-scores at 1 and 2 years of age [39]. Thus, the full impact of birth weight on development might become more evident in subsequent assessments.

The GA distribution showed that most infants in both groups were very preterm, with no significant differences observed between the groups. In comparison to the no-risk group, the higher risk group had a higher percentage of infants who were very preterm (21.9% vs. 78.1%). This finding implies that while GA is an important factor, other variables such as environmental influences may play a critical role in developmental outcomes. Recent research has shown that even moderate and late preterm births are associated with increased risks of DD and cognitive impairments compared with term births, although the magnitude of risk decreases with increasing GA [40,41]. Yet, the full extent of these risks may not be evident at 4 months CA, as developmental trajectories can evolve over time. Notably, the current cohort included four infants all from the high-risk group, where all had unresolved RSD that later developed into CLD. There is a strong association between CLD and a high risk of DD in infants and toddlers [42,43].

Despite the strengths of our study, including the use of a well-validated screening tool and a clearly defined sample, certain limitations should be acknowledged. Our sample size was relatively small, which may have limited the generalizability of our findings. Furthermore, the study did not include all infants that were born, which might restrict the feasibility of the study. The current study did not use a control group of full-term born infants to compare the findings of DD; this impacts the generalizability and limits the interpretation of our findings regarding the A-ASQ-3’s utility as a screening tool. While the study’s longitudinal design allowed for follow-ups, the A-ASQ-3 was only completed one time, which only gave a snapshot of the infant’s development at a specific time which may not permit causal inferences. The use of the non-probability convenience sampling risk may produce a selection bias by enrolling more interested parents and missing potential at-risk infants. Even though most parents completed the A-ASQ-3 on their own, assistance was provided when parents were not sure about an item in the A-ASQ-3; this, however, can impact the reliability of the scores. Another limitation of the study is the lack of comparison between the A-ASQ-3 findings and the gold-standard assessments such as the General Movement Assessment (GMA), Hammersmith Infant Neurological Examination (HINE), or Bayley Scales of Infant Development-III, thus impacting the validation the study’s result. While these tools serve as a reference standard for detecting developmental delays, their use was not feasible in the current study due to several constraints, including the availability of resources, time, and lack of certified therapists who are trained in GMA and HINE. For example, a recent study in Saudi Arabia found that only 16% of therapists are certified in using GMA [9], thus limiting the use of gold-standard assessments. Future studies should include larger samples, with a control group of full-term infants, and the use of standard tools for objective comparison. Moreover, more attention should be directed in training therapists to utilize gold-standard assessments in the field of early identification of DDs. In addition, policy and guidelines are required to unify practice in selecting early assessments to illustrate when and what type of assessment can be used.

Building on our current findings, we plan to conduct a longitudinal follow-up of preterm infants up to 18 months CA. This extended study will employ the ASQ-3 at multiple timepoints (4, 6, 9, 12, and 18 months) to track developmental trajectories and identify critical periods for intervention. This approach aligns with the recommendations of Schonhaut et al. for including the ASQ-3 in follow-up programs for children with biological risk factors such as preterm birth [28]. The current study showed that the A-ASQ-3 can be feasibly incorporated in future guidelines of Arabic-speaking countries as a screening tool where results from the screening can identify infants/toddlers at risk for further standardized assessments based on the domain.

## 5. Conclusions

In conclusion, our feasibility study highlights the critical need for early screening and tailored intervention programs for preterm infants in Saudi Arabia, setting the stage for more comprehensive longitudinal research in this region. Screening tools such as the A-ASQ-3 can be instrumental in identifying infants at high risk of DDs and warrant a formal standardized assessment, thereby improving the healthcare referral system.

## Figures and Tables

**Table 1:** Neonatal characteristics of preterm infants presented as frequency and percentage.

Neonatal Characteristics		All Infants (N = 48)	Infants with No Risk of DDs (N = 11)	Infants with High Risk of DDs (N = 37)	*p*-Value
Corrected age at assessment	12 weeks	34 (70.1)	8 (23.5)	26 (76.5)	
	13 weeks	14 (29.1)	3 (21.4)	11 (78.6)	0.9
Gender	Male	22 (45.8)	6 (27.3)	16 (72.7)	
	Female	26 (54.2)	5 (19.2)	21 (80.8)	0.4
Weight (g)	ELBW	7 (14.6)	4 (57.1)	3 (42.9)	
	VLBW	27 (56.2)	4 (14.8)	23 (85.2)	0.06
	LBW	14 (29.2)	3 (21.4)	11 (78.6)	
Gestational age (weeks)	Extremely preterm	8 (16.7)	3 (37.5)	5 (62.5)	
	Very preterm	32 (66.6)	7 (21.9)	25 (78.1)	0.5
	Moderate to late preterm	8 (16.7)	1 (12.5)	7 (87.5)	
Length of stay (days)		42.1 (23.4) ^†^	52.3 (20.4) ^†^	39.5 (24.0) ^†^	0.13
Respiratory distress syndrome	No	4 (8.3)	0	4 (100)	
	Yes	1 (2.1)	0	1 (100)	0.7
	Resolved	43 (89.6)	11 (25.6)	32 (74.4)	
Bronchopulmonary distress	No	44 (91.6)	10 (22.7)	34 (77.3)	0.9
	Yes	4 (8.3)	1 (25)	3 (75)	
Periventricular leukomalacia	No	43 (89.6)	10 (23.3)	33 (76.3)	0.8
	Yes	5 (10.4)	1 (20)	4 (80)	
Intraventricular hemorrhage (grade I and II)	No	30 (62.5)	5 (16.7)	25 (83.3)	
	Yes	18 (37.5)	6 (33.3)	12 (66.7)	0.2

^†^ Mean (SD); ELBW: extremely low birth weight; VLBW: very low birth weight; LBW: low birth weight.

**Table 2:** Results of the Ages and Stages Questionnaire for both the high-risk and no-risk infant groups.

A-ASQ-3 Domain	Infants with No Risk of DDs N (%)	Infants with High Risk of DDs N (%)	*P*-Value
Communication	41 (85.4)	7 (14.6)	0.18
Gross motor	31(64.6)	17 (35.4)	0.004 *
Fine motor	24 (50)	24 (50)	<0.001 *
Problem-solving	28 (58.3)	20 (41.6)	<0.001 *
Personal–social	17 (35.4)	31 (64.6)	<0.001 *

* Significant difference.

**Table 3:** Risk factors associated with high risk of developmental delays.

Risk of DDs	Odds Ratio	95% CI	*p*-Value
LOS	0.98	0.94–1.03	0.6
RDS	0.00	0.0–0.1	0.9
Weight at birth	1.0	0.99–1.03	0.6

LOS: length of stay; RDS: respiratory distress syndrome.

## Data Availability

The original contributions presented in this study are included in the article/Supplementary Materials. Further inquiries can be directed to the corresponding authors.

## Supplementary Materials

The following supporting information can be downloaded at: https://www.mdpi.com/article/10.3390/pediatric17050105/s1, Table S1: Results of the Ages and Stages Questionnaire for both the high-risk and no-risk infant groups.

## Abbreviations

The following abbreviations are used in this manuscript:

DD	developmental delays
ASQ	Ages and Stages Questionnaire
CA	corrected age
GA	gestational age
IVH	intraventricular hemorrhage
RDS	respiratory distress syndrome
CLD	Chronic Lung Disease
PVL	periventricular leukomalacia

## References

[1] Walani S.R. (2020). Global burden of preterm birth. Int. J. Gynecol. Obstet..

[2] Blencowe H., Cousens S., Oestergaard M.Z., Chou D., Moller A.B., Narwal R., Adler A., Vera Garcia C., Rohde S., Say L. (2012). National, regional, and worldwide estimates of preterm birth rates in the year 2010 with time trends since 1990 for selected countries: A systematic analysis and implications. Lancet.

[3] Fayed A., Wahabi H.A., Esmaeil S., Elmorshedy H., AlAniezy H. (2022). Preterm, early term, and post-term infants from Riyadh mother and baby multicenter cohort study: The cohort profile. Front. Public Health.

[4] Wahabi H., Fayed A., Esmaeil S., Alzeidan R., Elawad M., Tabassum R., Hansoti S., Magzoup M.E., Al-Kadri H., Elsherif E. (2016). Riyadh Mother and Baby Multicenter Cohort Study: The Cohort Profile. PLoS ONE.

[5] World Health Organization (2004). International Statistical Classification of Diseases and Related Health Problems: Alphabetical Index.

[6] Quinn J.A., Munoz F.M., Gonik B., Frau L., Cutland C., Mallett-Moore T., Kissou A., Wittke F., Das M., Nunes T. (2016). Preterm birth: Case definition & guidelines for data collection, analysis, and presentation of immunisation safety data. Vaccine.

[7] Spong C.Y. (2013). Defining “Term” Pregnancy: Recommendations From the Defining “Term” Pregnancy Workgroup. JAMA.

[8] Faruk T., King C., Muhit M., Islam M.K., Jahan I., Baset K.u., Badawi N., Khandaker G. (2020). Screening tools for early identification of children with developmental delay in low- and middle-income countries: A systematic review. BMJ Open.

[9] Gmmash A., Aljuhani T., Albesher R.A. (2025). Early Detection and Intervention Practices Provided by Physical and Occupational Therapists in Saudi Arabia for Children with or at Risk for Cerebral Palsy. J. Multidiscip. Healthc..

[10] Gmmash A.S., Faquih N.O. (2022). Perceptions of Healthcare Providers and Caregivers Regarding Procedures for Early Detection of Developmental Delays in Infants and Toddlers in Saudi Arabia. Children.

[11] Cheong J.L., Doyle L.W., Burnett A.C., Lee K.J., Walsh J.M., Potter C.R., Treyvaud K., Thompson D.K., Olsen J.E., Anderson P.J. (2017). Association Between Moderate and Late Preterm Birth and Neurodevelopment and Social-Emotional Development at Age 2 Years. JAMA Pediatr..

[12] Ryan M.A., Murray D.M., Dempsey E.M., Mathieson S.R., Livingstone V., Boylan G.B. (2023). Neurodevelopmental outcome of low-risk moderate to late preterm infants at 18 months. Front. Pediatr..

[13] Chen Z., Xiong C., Liu H., Duan J., Kang C., Yao C., Chen K., Chen Y., Liu Y., Liu M. (2022). Impact of early term and late preterm birth on infants’ neurodevelopment: Evidence from a cohort study in Wuhan, China. BMC Pediatr..

[14] Moeschler J.B., Shevell M. (2014). Comprehensive evaluation of the child with intellectual disability or global developmental delays. Pediatrics.

[15] Shatla M.M., Goweda R.A. (2020). Prevalence and Factors Associated with Developmental Delays among Preschool Children in Saudi Arabia. J. High Inst. Public Health.

[16] Lipkin P.H., Macias M.M. (2020). Promoting Optimal Development: Identifying Infants and Young Children With Developmental Disorders Through Developmental Surveillance and Screening. Pediatrics.

[17] Felix G., Deavenport-Saman A., Stavros S., Farboodi N., Durazo-Arvizu R., Garcia J., Yin L., Gera M.P. (2024). Standardizing and Improving Primary Care-Based Electronic Developmental Screening for Young Children in Federally Qualified Health Center Clinics. Matern. Child. Health J..

[18] Troude P., Squires J., L’Hélias L.F., Bouyer J., de La Rochebrochard E. (2011). Ages and Stages Questionnaires: Feasibility of postal surveys for child follow-up. Early Hum. Dev..

[19] Al-Hindi M.Y., Almahdi B.H., Alasmari D.A., Alwagdani R.K., Hunjur W.M., Khalel A.F., AlQurashi M.A. (2021). Screening for Neurodevelopmental Delay for Preterm Very Low Birth Weight Infants at Tertiary Care Center in Saudi Arabia. Cureus.

[20] Jain S., Patel P., Pandya N., Dave D., Deshpande T. (2023). Neurodevelopmental Outcomes in Preterm Babies: A 12-Month Observational Study. Cureus.

[21] Simard M.N., Luu T.M., Gosselin J. (2012). Concurrent validity of ages and stages questionnaires in preterm infants. Pediatrics.

[22] Schonhaut L., Pérez M., Armijo I., Maturana A. (2020). Comparison between Ages & Stages Questionnaire and Bayley Scales, to predict cognitive delay in school age. Early Hum. Dev..

[23] (2006). Identifying infants and young children with developmental disorders in the medical home: An algorithm for developmental surveillance and screening. Pediatrics.

[24] Vitrikas K., Savard D., Bucaj M. (2017). Developmental Delay: When and How to Screen. Am. Fam. Physician.

[25] Caesar R.A., Boyd R.N., Cioni G., Ware R.S., Doherty J., Jackson M.P., Salthouse K.L., Colditz P.B., PREMTiME Study Group (2023). Early detection of developmental delay in infants born very preterm or with very low birthweight. Dev. Med. Child Neurol..

[26] Moreira R.S., Magalhães L.C., Alves C.R.L. (2014). Effect of preterm birth on motor development, behavior, and school performance of school-age children: A systematic review. J. De Pediatr. (Versão Em Port.).

[27] Tanrıverdi M., Yılmaz G.G. (2025). Early neuromotor and sensory development in premature infants: An 18-month longitudinal follow-up study. Infant Behav. Dev..

[28] Schonhaut L., Armijo I., Schönstedt M., Alvarez J., Cordero M. (2013). Validity of the ages and stages questionnaires in term and preterm infants. Pediatrics.

[29] Charafeddine L., Sinno D., Ammous F., Yassin W., Al-Shaar L., Mikati M.A. (2013). Ages and stages questionnaires: Adaptation to an Arabic speaking population and cultural sensitivity. Eur. J. Paediatr. Neurol..

[30] Charafeddine L., Dani A., Badr L.K., Sinno D., Tamim H., Khoury J., Nasser F., Makki M. (2019). The psychometric properties of the Ages and Stages Questionnaires-3 in Arabic: Cross-sectional observational study. Early Hum. Dev..

[31] Saboktakin L. (2024). Developmental delay in preterm infants during the first twelve months after birth and its risk factors. J. Educ. Health Promot..

[32] Agarwal P.K., Shi L., Daniel L.M., Yang P.H., Khoo P.C., Quek B.H., Zheng Q., Rajadurai V.S. (2017). Prospective evaluation of the Ages and Stages Questionnaire 3rd Edition in very-low-birthweight infants. Dev. Med. Child Neurol..

[33] Habibullah H., Albradie R., Bashir S. (2019). Identifying pattern in global developmental delay children: A retrospective study at King Fahad specialist hospital, Dammam (Saudi Arabia). Pediatr. Rep..

[34] Valla L., Wentzel-Larsen T., Hofoss D., Slinning K. (2015). Prevalence of suspected developmental delays in early infancy: Results from a regional population-based longitudinal study. BMC Pediatr..

[35] Szatmari P. (2023). Editorial: The Importance of Screening for Developmental Disorders and Demonstrating Improved Health Outcomes. J. Am. Acad. Child. Adolesc. Psychiatry.

[36] Dorner R.A., Boss R.D., Burton V.J., Raja K., Lemmon M.E. (2020). Parent preferences for neurodevelopmental screening in the neonatal intensive care unit. Dev. Med. Child Neurol..

[37] Almalki N.S., Arrushaid O.M., Farah Bakhiet S., Alkathiri S. (2023). Examining the current practices of the individualized family services plan with young children with disabilities in Saudi Arabia. Int. J. Dev. Disabil..

[38] Duggan C., Irvine A.D., Hourihane J.O., Kiely M.E., Murray D.M. (2023). ASQ-3 and BSID-III’s concurrent validity and predictive ability of cognitive outcome at 5 years. Pediatr. Res..

[39] Nguyen P.T., Nguyen P.H., Tran L.M., Khuong L.Q., Nguyen S.V., Young M.F., Ramakrishnan U. (2024). Growth patterns of preterm and small for gestational age children during the first 10 years of life. Front. Nutr..

[40] Pettinger K.J., Copper C., Boyle E., Blower S., Hewitt C., Fraser L. (2023). Risk of Developmental Disorders in Children Born at 32 to 38 Weeks’ Gestation: A Meta-Analysis. Pediatrics.

[41] Song I.G. (2023). Neurodevelopmental outcomes of preterm infants. Clin. Exp. Pediatr..

[42] Laughon M., O’Shea M.T., Allred E.N., Bose C., Kuban K., Van Marter L.J., Ehrenkranz R.A., Leviton A. (2009). Chronic lung disease and developmental delay at 2 years of age in children born before 28 weeks’ gestation. Pediatrics.

[43] DeMauro S.B. (2021). Neurodevelopmental outcomes of infants with bronchopulmonary dysplasia. Pediatr. Pulmonol..

