# Supplementary material for: The Feasibility of the Arabic Version of Ages and Stages Questionnaire 3 to Identify Preterm Infants at Risk of Developmental Delays in Saudi Arabia
Source: Pediatr Rep. 2025 Oct 13;17(5):105. doi: 10.3390/pediatric17050105 (PMC12567042; doi:10.3390/pediatric17050105)
Supplement: Supplementary file 1 [file pediatrrep-17-00105-s001.zip › Table S1.pdf]

Table S1. Results of the Ages and Stages Questionnaire for both the high-risk and no-risk infant groups

A-ASQ-3 domain	Infants with no risk of DDs (N = 11) Mean (SD)	Infants with high risk of DDs (N = 37) Mean (SD)	P-value
Communication	54.9 (3.2)	41.7 (13.5)	0.10
Gross motor	52.2 (7.5)	34.7 (14.3)	0.004*
Fine motor	46.4 (9.3)	17.6 (15.2)	<0.001*
Problem-solving	56.2 (3.9)	26.7 (16.7)	<0.001*
Personal-social	42.9 (5.1)	17.9 (11.9)	<0.001*
